# Genetic characterization of Libyan date palm resources by microsatellite markers

**DOI:** 10.1007/s13205-013-0116-6

**Published:** 2013-02-05

**Authors:** M. L. Racchi, A. Bove, A. Turchi, G. Bashir, M. Battaglia, A. Camussi

**Affiliations:** 1 Genetics Section, Department of Agricultural Biotechnology, University of Florence, Via Maragliano 77, 50144 Florence, Italy; 2 Department of Plant Protection, Faculty of Agriculture, El Fatah University, Tripoli, Libya; 3 IAO-MAE, Via Cocchi 4, 50131 Florence, Italy

**Keywords:** Date palm, SSR, Identification, Genetic diversity, Cultivars

## Abstract

Molecular typing of 377 female date palm trees belonging to 18 Libyan cultivars and representing common genotypes in the central Libyan oasis of Al Jufrah was performed using 16 highly polymorphic microsatellite or SSR loci. A total of 110 alleles with an average of 6.88 alleles per locus were scored indicating the high level of polymorphism existing among the cultivars thus allowing their genetic fingerprinting. Moreover 28 alleles out of 110 were fixed. All the cultivars were characterized by negative values of the Fixation Index (*F*) due to an excess of heterozygotes with respect to HW equilibrium. The pattern of genetic diversity among cultivars was estimated by codominant genetic distances and presented by principal coordinates analysis (PCoA). The observed pattern evidences the genetic diversity existing among cultivars that allow distinguishing them easily. The average dissimilarity internal to each cultivar ranged from 0 to 21. Seven cultivars showed value zero indicating no genetic difference within cultivar in agreement with their Fixation Index (*F* = 1). A varietal identification key was also built using multiloci genotyping with only three microsatellite loci that identified 23 alleles in total. The possibility to attribute the unknown male plant to a cultivar was also considered and male parentage analysis was performed. Fifty-five male plants out of 63 were assigned to a definite cultivar with high confidence level. The positive result obtained in identifying males confirmed the suitability of SSR for clone fingerprinting and cultivar identification, thus opening new prospects for date palm breeding.

## Introduction

**Fig. 1:**
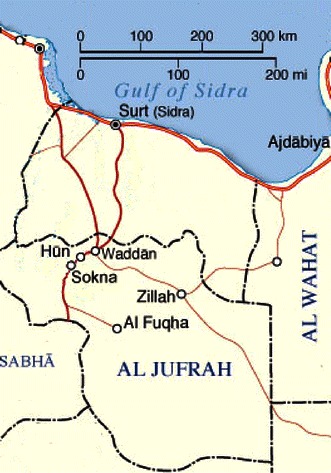
Al Jufrah area in Libya

Date palm (*Phoenix dactylifera* L., 2*n* = 36) is a perennial monocotyledonous fruit plant, belonging to the family of Arecaceae (Coryphoideae) (Barrow 1998, 1999). The genome size is estimated to be approximately 658-Mbp long (Al-Dous et al. 2011). Date is the most important fruit crop of arid climate region in North African and Middle East. Unlike other North African countries, in which the predominance of elite cultivars determined severe genetic erosion, date palm germplasm in Libya still preserves an enormous richness. More than 400 different date varieties are still grown in the country, out of which 95 are of commercial interest. This incredible richness has served as a highly effective natural defence for the Libyan plantations, which have remained safe from pathogens such as Bayoud disease and deserves to be preserved and evaluated in term of the genetic diversity. Dates can be classified based on the morphological and fruit features at harvest. Libya’s date varieties can be divided into three major groups: the fleshy-fruited coastal varieties, the semi-soft varieties from the central zone and the less succulent varieties, from the southern oases. The Al Jufrah oasis, located on 29th parallel and consisting of five localities, Hun, Waddan, Sokna, Zellah and Al Fugha, was chosen for sampling because it represents one of the most interesting Libyan regions for date palm cultivation (Fig. 1). In Libya, each palm grove is typified by a distinct cultivar composition, which results from a local selection within the oases. Date palms have been mainly clonally propagated by offshoots, just in few occasions seed propagation is performed using the pollen available from male trees of undefined origin. In general each cultivar derives from a unique descended individual of seed, cloned thereafter by vegetative multiplication to ensure the identity and uniformity of cultivars; however, intra-cultivar variation could potentially cause errors in cultivar identification. The demonstration of the true-to-type character for the plants is an important part of the quality assurance and it requires the use of markers effective in distinguishing the cultivars. Morphological traits and isoenzyme markers have been used in the past to describe and identify the date palm varieties of North Africa. Identification of date palm cultivars is principally based on morphology of leaves, spines and fruit characters. However, morphological traits are often variable or imprecise indicators of plant genotype, being influenced by environmental conditions or varying with the developmental stage of plant (Elhoumaizi et al. 2002). Furthermore biochemical approaches, including isoenzyme analysis, have been used to characterize North African date palms but such biochemical markers are limiting due to low levels of polymorphism (Al-Jibouri and Adham 1990) and discrimination among closely related cultivars and clones based on morphometric descriptors is often difficult. Nowadays, molecular markers, based on polymorphisms at DNA level, are increasingly used and proved effective to assess genetic diversity. Data based on molecular markers such as Random Amplified Polymorphic DNA (RAPD), Amplified Fragment Length Polymorphism (AFLP) and Restriction Fragment Length Polymorphism (RFLPs), have been used to characterize date palm genotypes. RAPD fingerprints have been used for identifying date palm accessions in Saudi Arabia (Al-Khalifah and Askari 2003), Egypt (Soliman et al. 2003; Adawy et al. 2005), Tunisia (Trifi et al. 2000) and Morocco (Sedra et al. 1998). Nevertheless these markers were not always successful in identification of date palm cultivars. Other markers as AFLP were used to reveal polymorphisms among date palm cultivars from Egypt and California (Cao and Chao 2002; El-Assar et al. 2003, 2005). To obtain a deeper comprehension of the genetic organization in date-palm germplasm other molecular markers were considered. For example, among molecular markers, microsatellites, also known as Simple-Sequence Repeats (SSRs), because of their particular features such as their co-dominant nature and their typically high levels of allelic diversity at different loci, represent a suitable tool for genotyping. The usefulness of microsatellite markers for measuring the genetic variability in a wide range of plants has been recently reviewed (Kalia et al. 2011). Because of their high mutation rates and the ease of the analysis microsatellite markers were proved useful and effective for phylogenetic studies (Pintaud et al. 2010), genetic fingerprinting and cultivar identification among different date palm accessions in California (Johnson et al. 2009), Tunisia (Zehdi et al. 2004a, b), Sudan (Elshibli and Korpelainen 2008), Oman (Al-Ruqaishi et al. 2008), Iraq (Khierallah et al. 2011) and Qatar (Ahmed and Al-Qaradawi 2009, Elmeer et al. 2011). Recently, date palm genome was de novo sequenced and annotated (Al-Dous et al. 2011). Data coming from genome analysis are functional to date palm improvement by means of biotechnology, being the cultivars differentiation and prediction of the gender of immature trees, the two most immediate challenges. The identification of male plants before flowering, because of the long juvenile phase of the date palm, represents a major constraint to breeding programs. In an attempt to determine sex, SSR markers were used by Elmeer and Mattat (2012). A promising candidate marker for male sex detection was detected among SSR markers tested because of the exclusive occurrence in individual male samples of a heterozygous allele. So far the pollinators are chosen by farmers, mainly, according to their availability during the female flowering time without any knowledge about their genotype. Accordingly, another interesting point to improve the fruit yield and quality is represented by the possibility to identify the genotype of male plants used as pollinators.

In this paper, we report the first data on the genetic fingerprinting using SSR of 18 Libyan date palm cultivars of Al Jufrah oasis. The objectives were (a) to investigate the genetic diversity of Libyan date palm, (b) the genotyping of cultivars, and (c) the parentage analysis of pollinator plants for the attribution to a cultivar.

## Materials and methods

### Plant material

**Fig. 2:**
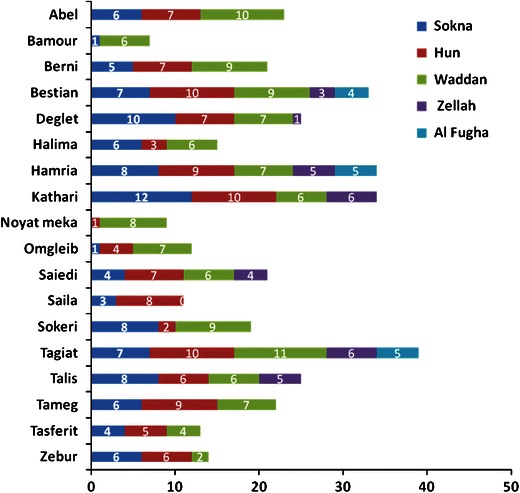
Distribution of cultivar samples among the localities of Al Jufrah oasis

Plant material consists of fresh leaves of 377 female trees belonging to 18 cultivars and of 63 male trees collected in date palm plantations, both from recent and ancient constitution in the localities of Sokna, Hun, Waddan, Zellah and Al Fugha (Fig. 1). The names of the commercially relevant cultivars sampled and the number of female trees sampled in each locality of Al Jufrah oasis are presented in Fig. 2. The samples were stored in paper bags at room temperature until DNA extraction.

### DNA extraction

The desiccated leaf material was ground into a fine powder using bead-mill homogenizer TissueLyser (Qiagen, Italy). The leaf powder of each individual sample was then subjected to DNA extraction using both DNeasy Plant Maxi/Mini Kits (Qiagen, Milano Italia) and E-Z 96 Plant kit (Omega), according to manufacturer’s instructions. The resulting DNA solutions were stored at –20 °C. After purification, DNA concentration was spectrophotometrically measured using Gene Quant Pro (Amersham Biosciences) and DNA quality was verified by visualization on a 1 % ethidium bromide agarose gel.

### Amplification and genotyping

A total of 16 date palms specific primer pairs were tested (Table 1). These were selected based on their polymorphic information content among SSR loci developed by Billotte et al. (2004) and Akkak et al. (2009). All the 377 samples were amplified and analyzed. PCRs were performed in a total reaction mixture of 14 μL containing 20 ng of total genomic DNA, 1× PCR buffer (Promega Corp. Madison, USA), 0.2 mM of dNTP (Promega), 0.05 U of Taq DNA polymerase (Promega), 0.07 μM of the forward primer with M13 tail, 0.2 μM of the reverse primer, and 0.2 μM of M13 primer-fluorescent dye (Invitrogen).

**Table 1:** Primer sequences of microsatellite loci used for genotyping of Libyan date palm cultivars

Source	Locus code	Primer sequences	
Billotte et al. (2004)	mPdCIR10	F:ACCCCGGACGTGAGGTG	R:CGTCGATCTCCTCCTTTGTCTC
	mPdCIR15	F:AGCTGGCTCCTCCCTTCTTA	R:GCTCGGTTGGACTTGTTCT
	mPdCIR25	F:CAAATCTTTGCCGTGAG	R:GGTGTGGAGTAATCATGTAGTAG
	mPdCIR32	F:CAAGACCCAAGGCTAAC	R:GGAGGTGGCTTTGTAGTAT
	mPdCIR70	F:CCATTTATCATTCCCTCTCTTG	R:CTTGGTAGCTGCGTTTCTTG
	mPdCIR78	F:CCCCTCATTAGGATTCTAC	R:GCACGAGAAGGCTTATAGT
	mPdCIR85	F:TGGATTTCCATTGTGAG	R:CCCGAAGAGACGCTATT
	mPdCIR93	F:GAGAGAGGGTGGTGTTATT	R:TTCATCCAGAACCACAGTA
Akkak et al. (2009)	PDCAT1	F:CTGAAATCTCTGTTCAAATCC	R:AGTTTGGATCTATTTGTGAGTTATTTTCTTT
	PDCAT2	F:GGCCTTCTCTTCCCTAATGGG	R:AGTTTCTTGCCCCTGTTCTTTC
	PDCAT6	F:AATCAGGGAAACCACAGCCA	R:GTTTAAAGCCTTCTCAAGATAGCCTCAG
	PDCAT8	F:GCTTAAGTGGTTAGTTGCCAA	R:GTTTGGCAGAAGTATTGAAAAGTTGA
	PDCAT11	F:TTAGTAGACTCCCCACCGTCC	R:TGTTTCATGGTGCTGGAGAATGAA
	PDCAT14	F:TGTTGCCATTCACATGCTGCG	R:TTTGGACTAGTCCCTCCCTCCC
	PDCAT17	F:TGCTGCAAATCTAGGTCACGAG	R:TTTACCCCTCGGCCAAATGTAA
	PDCAT18	F:CAGCGGAGGGTGGGCCTCGTT	R:TCTCCATCTCCCTTTTTCTTCTGCTACTC

For a given locus, the forward SSR primer was 5*′*-end labeled with M13 extension (5′-TGTAAAACGACGGCCAGT-3′) to incorporate, via PCR step, a fluorochrome (6-FAM, VIC and NED) necessary for the detection of the PCR products on the sequencer.

Amplifications were performed in Applied Biosystem Thermocycler (AB System, Germany) with the following conditions: for Billote’s primer, a initial denaturation at 95 °C for 1 min, then 35 cycles at 94 °C for 30 s, 52 °C for 1 min and 72 °C for 2 min and a final elongation step at 72 °C for 8 min; for Akkak’s primer a initial denaturation at 95 °C for 9 min, then 28–35 cycles at 94 °C for 30 s, 55 °C for 45 s and 72 °C for 1 min and a final elongation step at 72 °C for 45 min. A negative control, with the reaction mixture excluding DNA, was also included in each experiment.

Amplification products were checked on 1.5 % agarose gel to verify the presence of a band of the expected size. PCR products labeled with various fluorescent dyes (6′-FAM, NED and VIC) were loaded on the capillary system sequencer, MegaBACE1000 (GE Healthcare), with the size standard MegaBACE ET400 (GE Healthcare). Peaks were analyzed and fragment lengths determined with the MegaBACE Fragment Profiler 2.1 software (GE Healthcare). All peaks and binning were manually checked.

### Data scoring and genetic variability description

The observed heterozygosity (*H*_obs_) was calculated as the ratio of the number of heterozygotes and the number of samples for each locus and as arithmetic average over loci. The expected heterozygosity (*H*_exp_) assuming Hardy–Weinberg equilibrium was estimated as  where *p*_*i*_ is the frequency of the*i*-allele. The statistic was computed for single locus and as average over loci. The Unbiased Expected Heterozygosity (*UH*_exp_) takes into account the sample size *n*: .

The Fixation Index or Inbreeding Coefficient (*F*) was computed as .

Values close to zero are expected under random mating, while substantial positive values indicate inbreeding or undetected null alleles. Negative values indicate an excess of heterozygotes, due to negative assortative mating or selection (Hartl and Clark 1997).

The number of effective alleles (*N*_e_) provides an estimate of the number of equally frequent alleles in an ideal population with homozygosity equivalent to the actual population and was computed as .

### Distance metrics

The calculation of individual by individual Genetic Distance (GD) for SSR followed the method explained in Smouse and Peakall (1999). For the analysis of a SSR single-locus, first step involves the vectors calculation by additive genotype scoring convention per individuals (see Peakall et al. 1995). Subsequently, the squared distance () between any two genotypes is one-half the Euclidean distance between their respective pair of vectors as follows:where *i* and *j* are the genotypes and *k* is the scoring character. Squared distances range from zero, when individuals share the same alleles, to four when individuals are homozygous for different alleles. Genetic distance matrices for each locus were summed across loci under the assumption of independence.

The matrix of distances among cultivars is obtained as average of the individual distances between couple of cultivars, while the elements of the main diagonal are the average dissimilarities for all pair-wise comparisons internal to each cultivar. The off diagonal elements were submitted to Principal Coordinate analysis (covariance standardized method) and sample Eigenvectors were used to plot the cultivar centroids.

### Parentage analysis

Male trees were assigned to cultivars by a maximum-likelihood paternity assignment procedure through comparing genotypes of males and cultivars. To find the significant values of LOD scores, simulations were performed with 10,000 repeats, 0.01 as the proportion of loci mistyped and 61 individual profiles as probable cultivar candidate for each male tree. 95 % was used as strict and 80 % as relaxed confidence level as suggested by Marshall et al. (1998). The LOD score is obtained taking the natural log (log to base e) of the overall likelihood ratio. A positive LOD score means that the candidate variety is more likely to be the true variety. Negative load score can occur, when the candidate variety and query male tree share very common alleles or mismatch at one or more loci. A statistical test is performed on the base of the simulation of parentage analysis that allows determining also the confidence of parentage assignments.

Genetic variability measures and distance metrics were analyzed using GenAlEx 6.5 (Genetic Analysis in Excel; Peakall 2006; Peakall and Smouse 2012), available at: http://www.anu.edu.au/BoZo/GenAlEx. Cervus 3.0 (Kalinowski et al. 2007), available at http://www.fieldgenetics.com, was used for parentage analysis.

## Results

### Genetic diversity analysis

In a preliminary analysis 18 microsatellite primer pairs were tested, out of which the 16 more polymorphic and successfully amplified in all the samples were selected to perform the analysis to establish the genotypes of the 18 cultivars. Genetic diversity evaluated as number of alleles and genotypes for each SSR locus was presented in Table 2. A total of 110 alleles with an average of 6.88 alleles per locus were scored. The number of alleles per locus ranged from four for locus PDCAT1 to eleven for locus mPDCIR78; *H*_exp_ values ranged from 0.46 (mPdCIR 10) to 0.85 (mPdCIR78 and mPdCIR85) indicating that the Libyan date palm germoplasm is characterized by a high degree of genetic diversity. Moreover, 28 alleles out of 110 were fixed. Interestingly all the alleles at PDCAT1 locus were fixed even in different cultivars (Table 2). All 120 pair-wise comparisons among the 16 SSR loci did not show significant linkage disequilibrium.

**Table 2:** Summary of microsatellite allele data revealed by 16 microsatellites loci in 377 female trees belonging to 18 Libyan date palm cultivars

SSR locus	Allelic range (bp)	Total alleles	Number of genotypes	*H*_obs_	*H*_exp_	Number of fixed alleles	Fixed alleles	Cultivars^a^ with the fixed allele
mPdCIR 10	138–176	6	13	0.41	0.46	1	154	A, Be, D, Hal, O, S, Tag, Tam
mPdCIR 15	142–157	5	15	0.87	0.77	2	142	Tag
							157	Z
mPdCIR 25	219–257	6	17	0.90	0.76	1	249	Be
mPdCIR 32	306–321	5	13	0.71	0.66	2	316	S, Z
							309	Tal
mPdCIR 70	205–227	9	32	0.91	0.83	1	213	D
mPdCIR 78	126–173	11	36	0.85	0.85	2	136	Tag
							153	S
mPdCIR 85	175–199	8	39	0.83	0.85	2	175	O
							181	Hal
mPdCIR 93	181–197	7	17	0.77	0.77	1	197	O
PDCAT 1	103–123	4	10	0.23	0.63	4	103	Tam, Z
							105	Bes, S
							119	A, D, Hal, S
							123	Tal
PDCAT 2	186–209	7	20	0.85	0.79	2	186 206	Z
								Bes
PDCAT 6	142–172	7	17	0.82	0.71	2	150	Z
							158	O
PDCAT 8	222–258	6	14	0.78	0.68	2	246	S
							252	Tal
PDCAT 11	154–177	6	20	0.75	0.79	2	154	Z
							159	S
PDCAT 14	141–163	9	20	0.42	0.63	1	155	D, Hal, O, Tag, Tal, Tam
PDCAT 17	131–157	6	14	0.45	0.63	3	141	S
							147	A, D, K, N, O
							157	Tal
PDCAT 18	123–149	8	29	0.88	0.77	0	0	
Total		110				28		

*H*_obs_ observed heterozygosity by locus, *H*_exp_ expected heterozygosity at the HW equilibrium

^a^*A* Abel, *Ba* Bamour, *Ber* Berni, *Bes* Bestian, *D* Deglet, *Hal* Halima, *Ham* Hamria, *K* Kathari, *N* Noyat Meka, *O* Omglaib, *S* Saiedi, *Sa* Saila, *So* Sokeri, *Tag* Tagiat, *Tal* Talis, *Tam* Tameg, *Tas* Tasferit, *Z* Zebur

**Fig. 3:**
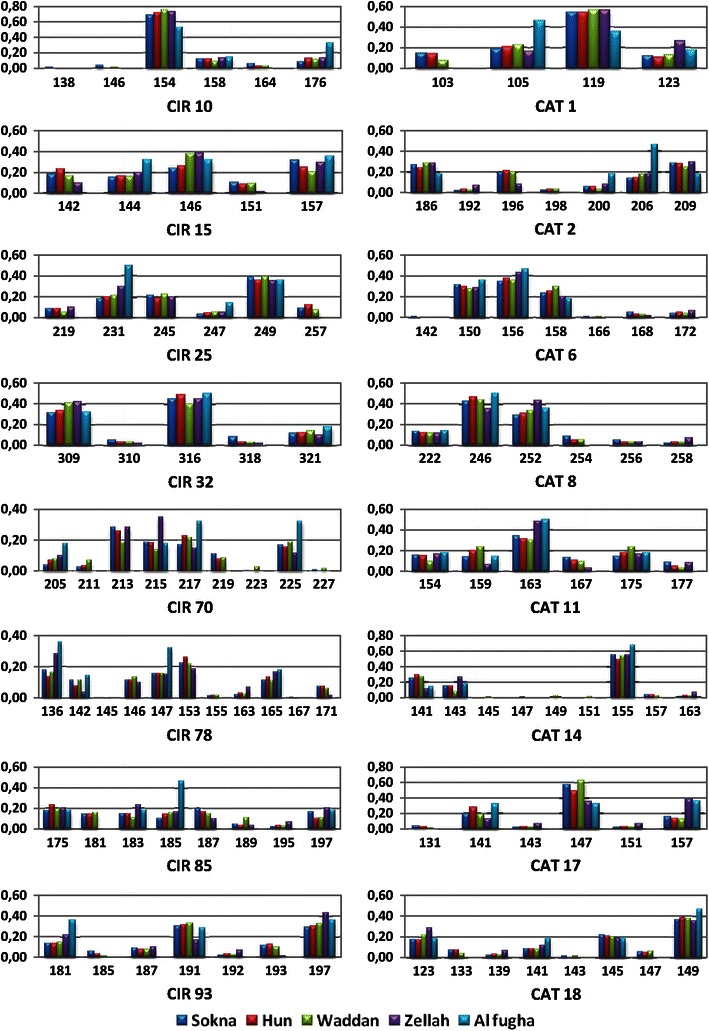
Microsatellite allele frequency distribution revealed by 16 microsatellites loci in the five localities of Al Jufrah oasis

The distribution of allele frequencies for the SSR loci in the five locality of Al-Jufrah are presented in Fig. 3. Both allele number and frequencies vary among the localities due to a different presence of the cultivars in the localities. A good example is represented by locus mPdCIR10, which exhibits six alleles: three of them were not present in all the localities (Fig. 2). The mean number of alleles varied from one cultivar to another (Table 3). The results put in evidence the different genetic structure of the cultivars. All the cultivars were characterized by negative values of the Fixation Index (*F*) due to an excess of heterozygotes with respect to HW equilibrium, though at different level. In particular, Talis, Halima, Omglaib, Saiedi, Tagiat, Saila and Zebur show *F* = −1, which indicates a strong heterotic selection at the base of the clonal breeding of these cultivars. On the other hand an *F* value close to zero is expected under random mating, as observed in Sokeri that is traditionally seed propagated. The average percentage of polymorphic loci was 81. Bamour, Hamria, Sokeri and Tasferit resulted polymorphic at all the loci. The smallest percentage (56.25) was shown by the cultivar Omglaib (Table 3).

**Table 3:** Genetic diversity indices for the 18 Libyan date palm cultivars

Cultivar	*N*	*N*_a_	*N*_e_	*H*_obs_	*H*_exp_	*UH*_exp_	*F*	*P* %
Abel	23	1.938	1.767	0.747	0.379	0.387	−0.973	75.00
Bamour	7	2.813	2.121	0.653	0.491	0.530	−0.329	100.00
Berni	21	2.625	1.733	0.622	0.348	0.356	−0.790	87.50
Bestian	33	2.750	1.684	0.629	0.339	0.344	−0.855	93.75
Deglet	25	1.688	1.630	0.625	0.314	0.320	−0.992	62.50
Halima	15	1.750	1.750	0.750	0.375	0.388	−1.000	75.00
Hamria	34	3.313	2.049	0.915	0.497	0.504	−0.842	100.00
Kathari	34	2.500	1.861	0.807	0.425	0.432	−0.897	93.75
Noyat Meka	9	2.563	1.755	0.576	0.365	0.387	−0.578	93.75
Omglaib	12	1.563	1.563	0.563	0.281	0.293	−1.000	56.25
Saiedi	21	1.813	1.813	0.813	0.406	0.416	−1.000	81.25
Saila	11	1.625	1.625	0.625	0.313	0.327	−1.000	62.50
Sokeri	19	5.125	3.630	0.708	0.656	0.674	−0.079	100.00
Tagiat	39	1.813	1.813	0.813	0.406	0.412	−1.000	81.25
Talis	25	1.625	1.625	0.625	0.313	0.319	−1.000	62.50
Tameg	22	2.750	1.833	0.685	0.380	0.389	−0.802	81.25
Tasferit	13	3.938	2.124	0.630	0.478	0.497	−0.319	100.00
Zebur	14	1.625	1.625	0.625	0.313	0.324	−1.000	62.50
Mean	20.9	2.434	1.889	0.689	0.393	0.406	−0.803	81.60
SE	0.5	0.071	0.043	0.025	0.014	0.014	0.028	3.63

*N* sample size, *N*_a_ number of alleles, *N*_e_ number of effective alleles, *H*_obs_ observed heterozygosity, *H*_exp_ expected heterozygosity at the HW equilibrium, *UH*_e_ unbiased expected heterozygosity, *F* Fixation Index, *P* % percentage of polymorphic loci

**Fig. 4:**
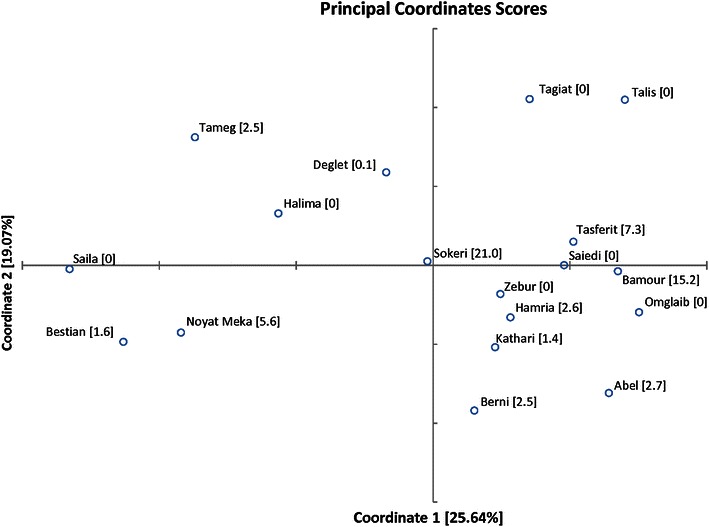
Score plot of Libyan date palm cultivars on the first two principal coordinates from codominant genotypic distances (Smouse and Peakall 1999) of 16 microsatellite loci. The average dissimilarity for all pair-wise comparison internal to each variety is reported in bracket

The pattern of genetic relationships contained in the matrix of codominant genotypic distances of SSR loci is presented in Fig. 4 by plotting the cultivars on the first two axes of principal coordinate analysis (PCoA). Thought the first two axes explain a cumulative percentage of variation of 44.7, the plotted pattern evidences the genetic diversity existing among cultivars that allows distinguishing them easily. Codominant genotypic distances enable estimating also the average dissimilarity internal to each cultivar (in bracket in Fig. 4) ranging from 0 to 20.98. Talis, Halima, Omglaib, Saiedi, Tagiat, Saila and Zebur showed value zero, indicating no genetic difference within cultivar in agreement with the fixation index reported in Table 3. The highest value observed for Sokeri (21.0) is expected on the base of the breeding by seed that characterize the cultivar.

### Cultivar identification key

**Fig. 5:**
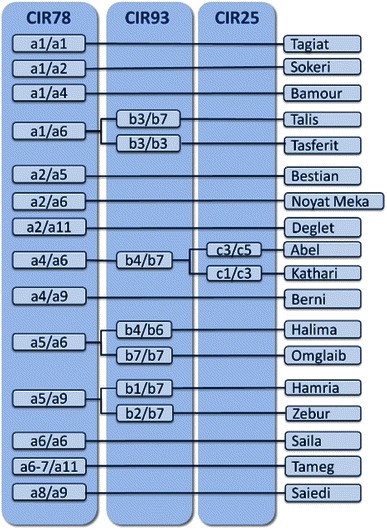
Identification key of 18 Libyan palm date cultivars based on three microsatellites locus fingerprints

For each date palm cultivar, the detected genotypes for mPdCIR78, mPdCIR93, mPdCIR25 microsatellite loci were scored. A total of 23 alleles were identified in these loci: ten alleles labeled (a1–a11) for mPdCIR78 locus, seven alleles (b1–b7) for mPdCIR93 locus and sux alleles (c1–c6) for mPdCIR25 locus. An identification key was established considering the informative allele combinations (Fig. 5). All the cultivars exhibited different fingerprints across all the SSR loci examined confirming the ability of these markers to fingerprint genotypes. Accordingly, the constructed identification key allowed to discriminate all the cultivars studied.

### Identification of male plants

Considering the SSR effectiveness in fingerprinting genotypes they were also used to assign to a cultivar the male plants sampled in each farm of the different localities of Al Jufrah oasis. The maximum-likelihood paternity assignment procedure used allowed to assign each male plant to a single cultivar: 55 out of 63 males were assigned to cultivars with strict confidence. Among them 24 males with positive LOD scores are listed in Table 4. Positive LOD scores range from 0.13 to 9.90. These 24 male plants were collected from farms of Sokna, Hun and Waddan and were assigned to nine cultivars (four to Bastian and Tagiat and three to Abel, Deglet, Sokery and Tasferit, respectively).

**Table 4:** Assignments with positive scores of pair LOD value of parentage analysis performed on male plants sampled in Al Jufrah farms

Collection site	Male code	Candidate cultivar	Pair LOD score
Sokna	SMM-Q-04	Kathari	9.47
	SME-Q-03	Tasferit	0.13
	SMT-Q-01	Tasferit	6.60
	SMT-Q-02	Sokeri	1.26
	SSA-Q-01	Deglet	9.87
	SSA-Q-02	Deglet	1.77
	SAK-Q-02	Bestian	6.97
	SAK-Q-03	Sokeri	2.20
Hun	HSM-Q-02	Bestian	1.26
	HRG-Q-01	Tagiat	3.08
	H6I-Q-02	Deglet	1.05
	H6I-Q-03	Tagiat	5.73
	H6E-Q-03	Bamour	1.33
	H3F-Q-03	Tameg	1.26
	H5H-Q-02	Abel	3.45
	H5H-Q-03	Hamria	2.16
	H3H-Q-02	Bestian	1.12
Waddan	WOE-Q-02	Abel	9.15
	WBH-Q-01	Abel	3.33
	WBH-Q-02	Sokeri	1.75
	WFZ-Q-03	Tagiat	9.90
	WHS-Q-01	Tagiat	3.80
	W4B-Q-03	Hamria	1.82
	WBB-B	Tasferit	1.60

## Discussion

Eighteen cultivars representing common genotypes in Al Jufrah oasis selected for their good fruit quality were analysed using 16 highly polymorphic microsatellite loci. Date palm germplasm of Libya still preserves an enormous richness attested by a great number of different accessions, widely described since the beginning of twentieth century on the base of the morphological characters of fruit and seeds. The date palm genetic resources deserve nowadays to be genetically characterized with the aim to organize their preservation, to transmit a significant genetic richness and for their exploitation. In Libya, each palm grove is typified by a distinct cultivar composition, which resulted from a local selection within the oases. As a consequence, a large number of SSR alleles have been revealed with a mean of 6.88 per locus that allowed detecting a relatively high degree of genetic variability in this crop. A high level of polymorphism was detected among cultivars as previously reported in date-palm cultivars of Algeria, Morocco, Tunisia and Sudan using both isoenzymes and SSR markers (Benaceur et al. 1991; Elhoumaizi et al. 2006; Zehdi et al. 2004a, b; Elshibli and Korpelainen 2008, 2009). Furthermore the presence of higher polymorphism within the date palm genome was evidenced by the results obtained from parallel sequencing (Al-Dous et al. 2011).

Each cultivar results from an empirical selection for heterosis carried out by the farmers based on morphological characters and fruit quality. This fact justifies both the presence of fixed alleles, 28 out of 110, due to random drift and the high level of heterozygosity maintained by clonal breeding procedure (Table 2).

For the analysis of the SSR multilocus genotypes the procedure of Smouse and Peakall (1999) was used. This method allows evaluating the genetic differences at different levels of structure, such as cultivar and locality, based on distances among the individual genetic profile of each sampled tree. The differences, if any, among plants of the same cultivars sampled in the different localities of Al Jufrah oasis were not significant (data not shown). In the case of cultivar Hamria, for example, the statistic PhiPT (*Φ*_PT_) receives the value of 0.091, which is not significant after 999 permutations and particularly in three localities out of five all the trees exhibited the same genotype.

The PCoA based on codominant genotypic distances of SSR loci (Fig. 4) represents a procedure by which the revealed genetic pattern does not assume a hierarchical structure like tree building methods consequently, it is appropriate for analysing our date palm samples, in which the assumption of a high taxonomic level is less reasonable. The results evidence the genetic diversity existing among cultivars that enable distinguishing them easily. Furthermore the codominant genetic distances procedure of Smouse and Peakall (1999) allows estimating the average dissimilarity internal to each cultivar. In seven cultivars out of eighteen absence of genetic diversity within cultivar was observed in agreement with the respective Fixation Indexes (Table 3).

The traditional practice of vegetative multiplication of plants by offshoots, which was generally performed with good skill by Al Jufrah farmers, ensures the identity and uniformity of the cultivars. Nevertheless, cases of misclassification can occur during propagation because of the difficulty faced sometimes in the identification of the cultivars on the base of morphology. The cultivar Sokeri represents an exception and seed propagation performed in some farms of Waddan resulted in the high distance values observed within this cultivar. Seed propagation of date palm has been reported to occur also in other countries. Elshibli and Korpelainen (2008) while analyzing Sudan date palm resource, because of high genetic diversity observed within groups and the weak clustering of the cultivars suggested that they are not a result of a full cloning process. The ease and rapidity of seed reproduction coupled with their large availability support the maintenance of this practice among Sudan farmers consequently, date palm plantations are a mixture of plants both clonally or seed propagated with a high genetic variability within cultivars. Clonal propagation, beyond to guarantee genetic uniformity of the cultivars, also limits the negative effect of inbreeding. In fact, it provides the maintenance of high level of heterozygosity within cultivars achieved by assorting heterotic positive characteristics as result of empirical selection of plants with good pomology features and fruit quality. The analysis of genetic variation between localities for each cultivar was performed and differences were not significant.

The strong cultivar genetic identity observed made possible to design an identification key based on a three microsatellite loci that identified 23 alleles in total and permitted the unambiguous discrimination of the date palm accessions. Consequently, the totality of Al Jufrah cultivars was univocally and easily identified on the base of their allelic profile. Similar result was previously obtained by Zehdi et al. (2006) in the analysis of 49 Tunisian accessions with three SSR loci. The effectiveness of SSR in discriminating all the accessions and cultivars examined confirms the usefulness of these markers for clonal fingerprinting and cultivar identification. Since each variety was identified by a unique profile, it is possible to generate an individual barcode useful in certification and control of origin labels of date palm products. On the contrary of other species of economic relevance, in the case of *Phoenix dactilifera* specific test guidelines of the UPOV system based on morphological descriptors are not requested (http://www.upov.int/test_guidelines/en). On the other hand, vegetative and fruiting traits are limited in number and influenced by the environmental conditions and their use can result in a poor discriminant power. Consequently, the introduction of molecular fingerprinting for variety protection and farmer rights is an important perspective to achieve also in the perspective of product origin certification for international trading.

The improvement of fruit yield and quality is based on the possibility to identify the genotype of male plants used as pollinators. Generally female plant pollination is carried out by mixing pollen coming from the few male plants present in the farm. Most of the time the cultivar identity of male plant is unknown because of seed propagation and the exchanges that often occur among farmers. Considering that genetic variability observed among cultivars is higher than that within cultivars, a full sib marker assisted selection procedure could be proposed starting from the cross of cultivars with different positive traits. For that purpose to attribute an unknown male tree to a cultivar becomes important. Consequently, a parentage analysis by a procedure of maximum-likelihood paternity assignment was performed through comparing genotypes of males and cultivars. The results obtained allowed to assign, with high confidence level, 55 male trees out of 63 to a distinct cultivar. Furthermore the identification key applied to the 24 male presenting positive LOD score evidenced that each male has at least one allele in common with the cultivar assigned by the parentage analysis. The methodology used do not require a priori identification of sex-specific SSR markers, but only the availability of a molecular profile representative of each candidate variety. The positive result obtained by assigning male trees further confirms the suitability of SSR for genotyping and opens new prospects for date palm breeding for yield and improved physical and chemical characteristics of the fruits.
